# Prevalence of myocardial viability in dysfunctional areas by cardiovascular magnetic resonance in patients with coronary artery disease

**DOI:** 10.1186/1532-429X-13-S1-P159

**Published:** 2011-02-02

**Authors:** Lara Bakhos, Maria Manuela Izquierdo-Gomez, Edwin Wu, Mihai Gheorghiade, Jeffrey J Goldeberger, Alan H Kadish, Daniel C Lee

**Affiliations:** 1 Northwestern University, Chicago, IL, USA

## Background

The presence of dysfunctional yet viable myocardium (DV-Myo) is known to predict functional recovery after revascularization and long-term prognosis. We sought to define the prevalence of DV-Myo by cardiovascular magnetic resonance imaging (CMR) in patients with coronary artery disease.

## Methods

We analyzed 181 patients from the DEfibrillators To REduce Risk by MagnetIc ResoNance Imaging Evaluation Trial. All patients underwent cine and contrast-enhanced (CE) CMR. Cine and CE studies were scored on a 17-segment model by the consensus of two readers. Cine images were scored for wall motion (WM): 0 = normal, 1 = mild hypokinesis, 2 = moderate to severe hypokinesis, 3 = akinesis, 4 = dyskinesis. CE images were scored for hyperenhanced (HE) infarct transmurality: 0 = none, 1 = 1-25% HE, 2 = 26-50% HE, 3 = 51-75% HE, 4 = 76-100% HE. Manually planimetered, quantitative analysis was also performed to obtain LV ejection fraction (EF) and infarct size using QMass MR 7.2 (Medis, Leiden, the Netherlands). DV-Myo segments were defined as having WM score ≥ 2 and HE score ≤ 1. DV-Myo involving 2-4 of 17 segments (12-24% of LV) was considered prognostically significant only. DV-Myo involving ≥ 5 of 17 segments (≥ 29% of LV) was considered functionally significant.

## Results

Baseline characteristics included male sex (84%) and mean age 61.5 ± 11.2 (31-88). Patients were on standard medical therapy, including beta-blockers (93%), ACE inhibitor/ARB (82%), anti-platelet (100%) and lipid lowering agents (94%). The population had a mean EF of 38.9 ± 11.6% (range 11.4 - 69.1%) and infarct size 17.3 ± 10.3% of LV (range 0 - 59.5%). A total of 3077 segments were evaluated, of which 442 (14%) segments met criteria for DV-Myo. Prognostically significant DV-Myo was present in 49/181 patients (27.1%). These patients had a mean of 2.7 ± 0.7 affected segments and a mean EF of 34.4 ± 7.7%. Functionally significant DV-Myo was present in an additional 33/181 patients (18.2%). These patients had a mean of 8.1 ± 3.0 affected segments and a mean EF of 26.2 ± 8.5%.

## Conclusions

A substantial proportion of patients with CAD have prognostically significant or functionally significant areas of DV-Myo. This highlights the need for viability assessment to guide appropriate therapy in these patients.

